# Genomic Differences Associated with Resistance and Virulence in *Pseudomonas aeruginosa* Isolates from Clinical and Environmental Sites

**DOI:** 10.3390/microorganisms12061116

**Published:** 2024-05-30

**Authors:** Kelly J. Aroca Molina, Sonia Jakeline Gutiérrez, Neyla Benítez-Campo, Adriana Correa

**Affiliations:** 1 Department of Biology, Faculty of Natural and Exact Sciences, Universidad del Valle, Cali 760042, Colombia; kelly.aroca@correounivalle.edu.co (K.J.A.M.); sonia.gutierrez@correounivalle.edu.co (S.J.G.); 2 Department of Basic Sciences, Universidad Santiago de Cali, Cali 760035, Colombia; adriana.correa00@usc.edu.co

**Keywords:** antibiotic resistance, comparative genome, *Pseudomonas aeruginosa*, virulence, whole-genome sequencing

## Abstract

*Pseudomonas* aeruginosa is a pathogen that causes healthcare-associated infections (HAIs) worldwide. It is unclear whether *P. aeruginosa* isolated from the natural environment has the same pathogenicity and antimicrobial resistance potential as clinical strains. In this study, virulence- and resistance-associated genes were compared in 14 genomic sequences of clinical and environmental isolates of *P. aeruginosa* using the VFDB, PATRIC, and CARD databases. All isolates were found to share 62% of virulence genes related to adhesion, motility, secretion systems, and quorum sensing and 72.9% of resistance genes related to efflux pumps and membrane permeability. Our results indicate that both types of isolates possess conserved genetic information associated with virulence and resistance mechanisms regardless of the source. However, none of the environmental isolates were associated with high-risk clones (HRCs). These clones (ST235 and ST111) were found only in clinical isolates, which have an impact on human medical epidemiology due to their ability to spread and persist, indicating a correlation between the clinical environment and increased virulence. The genomic variation and antibiotic susceptibility of environmental isolates of *P. aeruginosa* suggest potential biotechnological applications if obtained from sources that are under surveillance and investigation to limit the emergence and spread of antibiotic resistant strains

## 1. Introduction

*Pseudomonas aeruginosa* a Gram-negative bacterium of the Pseudomonadaceae family, exhibits exceptional adaptability and metabolic versatility [1]. It thrives in diverse habitats, including soil, water, plants, animals, hospitals, and wastewater, demonstrating ecological resilience [2]. This adaptability stems from its ability to mineralize organic matter and utilize diverse substrates, some of which are toxic, while also exhibiting resistance to heavy metals, antimicrobials, and detergents [3,4,5].

Additionally, it is known that in order to respond and adapt to different environments, *P. aeruginosa* has a repertoire of genes that are substantially conserved and that correspond to the highest proportion of genes and regulatory networks observed in known bacterial genomes [6]. The genomic of *P. aeruginosa*, referred to as the pangenome, comprises a core genome of conserved elements and an accessory genome of variable genetic material. This genetic duality contributes to its ability to respond and adapt to diverse environments. Notably, the core genome encodes shared metabolic and pathogenic factors, while the accessory genome, characterized by high G + C content and mobile genetic elements, showcases genetic plasticity [6,7].

This opportunistic bacterium is considered one of the main pathogens associated with healthcare-associated infections worldwide due both to its ability to persist under adverse environmental conditions and to the pathogenicity mechanisms it possesses [8]. This high pathogenicity is also closely associated with its great genetic plasticity, conferred by regulatory genes, coding genes for intrinsic and acquired resistance, which promote greater persistence and dissemination, preventing current antimicrobials from being effective in controlling infections caused by this bacterium [7]. Recognized as a significant pathogen in healthcare-associated infections globally, *P. aeruginosa* pathogenicity is linked to its genetic plasticity and regulatory genes governing intrinsic and acquired resistance. Clinical investigations reveal varying virulence among strains with the accessory genome playing a pivotal role in this heterogeneity [9].

The population structure of *P. aeruginosa* is nonclonal and epidemic. However, certain strains such as ST235 and ST111 are considered high-risk clones (HRCs) due to their global dissemination and association with multidrug resistance (MDR/XDR). These clones, particularly ST235, are associated with over 60 variant β-lactamases and high virulence due to ExoU positive cytotoxin, making them a growing concern in hospitals. Clones such as ST111 and ST233 are associated with the metallo-β-lactamase (MBL) VIM-2. ST244 is frequently found but not always multidrug resistant (MDR)/extensively drug resistant (XDR). ST357, ST308, and ST298 are also ExoU positive and potentially more virulent [10,11,12].

Furthermore, the ubiquity of the bacterium in clinical settings is due in part to its intrinsic and acquired antibiotic resistance, making it multidrug resistant or extensively drug resistant. It is unclear whether these characteristics persist in non-hospital settings; however, recent studies of *P. aeruginosa* highlight the prevalence of multidrug-resistant strains, particularly in wastewater [13,14].

Nevertheless, the potential pathogenicity and drug resistance of environmental strains remain uncertain and of concern if the biotechnological potential demonstrated by *P. aeruginosa* in environmental pollution mitigation, such as in bioremediation of oil spills, among other uses, is to be harnessed [15]. However, concerns about its pathogenicity highlight the need to know whether representatives of *P. aeruginosa* isolated from the out-of-hospital environment may differ in their genetic composition from those isolated from clinical environments such that they can be employed in biotechnological uses [5,16].

On the other hand, genomic analyses reveal that mutations and horizontal gene transfers contribute to the resistance arsenal [6,7,17,18]; likewise, advanced molecular techniques, including whole-genome sequencing, have improved our understanding of the epidemiology of *P. aeruginosa* [19,20]. In particular, whole-genome sequencing provides information on clonal dissemination, prevalence and global spread, especially with regard to antibiotic resistance determinants [12,21].

Therefore, in this research, we compared the genetic characteristics associated with the virulence and resistance of clinical and environmental isolates of *P. aeruginosa.*

## 2. Materials and Methods

### 2.1. Selection of Isolates

Seven clinical isolates and seven environmental isolates of *P. aeruginosa* were selected for this study. The clinical isolates came from patients with acute infections and were provided by the microorganism collection of the Santiago de Cali University, which were coded as 10, 11, 20, 23, 31, 37 and 46; environmental isolates were randomly selected from the collection of microorganisms of the Microbiological Research Laboratory of the Valle University (Cali, Colombia); their description is summarized in Table 1.

### 2.2. Antimicrobial Susceptibility Testing

All isolates were tested for susceptibility to aztreonam, ceftazidime, cefepime, ciprofloxacin, imipenem and meropenem, piperacillin/clavulanic acid and amikacin. The minimum inhibitory concentrations (MICs) of these antibiotics were determined by the broth microdilution method according to the Clinical & Laboratory Standards Institute (CLSI) 2024 breakpoints [22]. *P. aeruginosa* ATCC 27853 was used as a control. 

### 2.3. MLST Typing

The collection of isolates was typed using MLST (multilocus sequence typing), and the ST was calculated for each genome [23] submitted to the PubMLST database [24].

### 2.4. DNA Extraction and Sequencing

DNA extraction and sequencing of environmental and hospital isolates of *P. aeruginosa* were performed at the International Center for Microbial Genomics of the Bosque University (Bogotá, Colombia) using Illumina technology. Nextera XT genomic libraries were prepared from DNA extracted using the Qiagen^®^ (Hilden, Germany) DNeasy Blood & Tissue Kit according to the manufacturer’s instructions [25]. The quality, purity and concentration of the DNA were verified using Nanodrop^®^ and Qbic^®^ from Thermo Fisher^®^ (Waltham, MA, USA). Whole genome sequencing was performed using the MiSeq system, and paired sequences were obtained for each isolate with a read length of 2–149 bp [26]. Quality analysis of the raw sequences was performed on the Galaxy Europe platform using FastQC^®^ version 0.11.9 [27], which was followed by trimming and filtering using Trimmomatic^®^ version 0.38 [28].

### 2.5. Identification of Genes Associated with Virulence and Antimicrobial Resistance

Genome assemblies were screened for antimicrobial resistance (AMR) and virulence genes for possible correlation with phenotypic observations. The Unicycler^®^ program version 0.4.8 was used to individually assemble the sequences [29]. Annotation was performed using the Prokka^®^ server version 1.14.6 [30]. The PATRIC^®^ software version 3.6.12 was used to identify virulence and antibiotic resistance genes [31]. Virulence genes were identified using the VFDB database [32], while antibiotic resistance genes were identified using the PATRIC and CARD databases [33,34].

### 2.6. Comparison of the Genomes

Data obtained from the PATRIC, CARD and VFDB databases were organized in tables to compare the presence or absence of 239 virulence genes and 135 resistance genes detected among clinical and environmental isolates of *P. aeruginosa*. Statistical analysis was then performed using RStudio^®^ software (Version: 2023.12.1+402) using the generalized linear model (GLM) with binomial error distribution and an analysis of variance (ANOVA) to determine whether the differences found were statistically significant among the genes analyzed (*p* value < 0.05) [35,36,37]. The circular BLAST Ring Image Generator (BRIG^®^) tool was used, with 90% upper sequence identity and 70% lower sequence identity whose cutoff value was E 1 × 10^−5^ to show the presence or absence and relative location of virulence genes of the 14 *P. aeruginosa* isolates [38]. Finally, a heat map was created to display the differences in the presence and absence of antibiotic resistance-related genes.

## 3. Results

### 3.1. Antimicrobial Susceptibility Testing

According to CLSI breakpoints, five of the seven environmental isolates were found to be susceptible to all antibiotics tested. The isolate from sugarcane soil (s33) was resistant to aztreonam, and one isolate from DWTP (p3) was resistant to ciprofloxacin, amikacin, and aztreonam, considered as MDR. Out of the clinical isolates, only two were found to be susceptible to all antibiotics tested (10 and 11), one isolate was resistant to ciprofloxacin (20) and the remaining isolates were classified as MDR (23, 31, 37, and 46) (Table 2). Notably, according to the European Committee on Antimicrobial Susceptibility Testing (EUCAST), the susceptible isolates would be classified as resistant to aztreonam [39].

### 3.2. Multilocus Sequence Typing (MLST)

Determination of the sequence type (ST) of the isolates included in this study showed that none of the environmental isolates were associated with a high-risk clone (HRC). In contrast, STs associated with HRC were present exclusively in three clinically derived isolates, 37 and 46 are ST235, and 11 had ST111.

### 3.3. General Characteristics of Genomes

The genome length of the environmental isolates ranged from 6.2 to 6.5 Mbp, which is slightly smaller than that of the clinical isolates, which ranged from 6.4 to 7.0 Mbp. Note that the whole genome analysis was performed without separating the chromosome from the plasmid. Also, the analysis of the environmental isolates showed that they had smaller coding regions (CDs) compared with the clinical isolates (mean values of 5919.7 and 6430, respectively), while the G + C content was very similar among all isolates, ranging from 66.0 to 66.6% (Table 3). The sequence of all genomes was deposited in the NCBI GenBank database under the following bioproject SUB14219539 https://www.ncbi.nlm.nih.gov/sra/PRJNA1075801 (accessed on 12 February 2024).

### 3.4. Comparison of Genes Associated with Virulence

**Figure 1:**
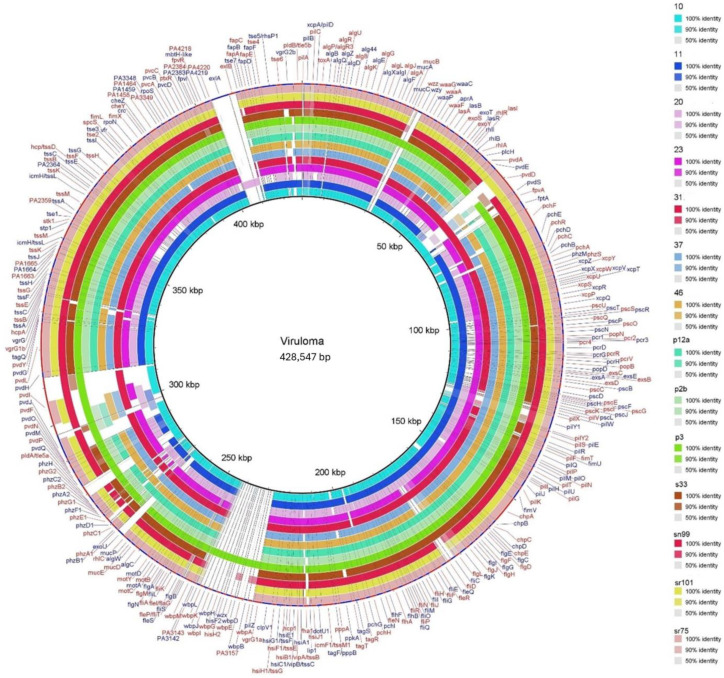
Circular map representing the virulome of the 14 *P. aeruginosa* isolates from environmental and clinical settings. Each circle on the map represents a genome with colors indicating the presence of genes and spaces indicating their absence. The outer rings (1–7) represent environmental isolates, while the inner rings (8–14) represent clinical isolates.

A total of 239 virulence-associated genes were detected in all *P. aeruginosa* isolates studied, and the virulome is represented in the circular BRIG map (Figure 1). The results showed that 149 genes (62.3%) were common to both clinical and environmental isolates. Among these common genes, 85 were related to adhesion and motility mechanisms, followed by 76 genes associated with type II, III, and VI secretion systems, and 46 genes associated with the quorum sensing mechanism (*lasI/lasR* and *rhlI/rhlR*), which were located between 10 and 50 kbp on the circular map (Figure 1 and Supplementary Material S1).

Genes related to the production of siderophores, which enable nutrient uptake and are involved in *P. aeruginosa* virulence [40], were observed to predominate in environmental isolates and were located between 50 and 90 kbp, 190 and 200 kbp, 270 and 320 kbp and 390 and 400 kbp.

In addition to this information, differences in the presence of the *exoU* gene, which codes for the protein of the same name and is associated with the type III secretion system (S3TT) required for bacterial cytotoxicity, were found, mainly in clinical isolates, between 260 and 270 kbp. The presence of the *exoU* gene was significantly different (*p* = 0.0475) in clinical isolates compared to those from the natural environment. In particular, clinical isolate 20 lacked SST3-associated genes, but it had the selection genes *exlA* and *exlB* (400 to 420 kbp), which encode the exlA and exlB toxins associated with hypervirulent strains [41], the gene responsible for rhamnolipid production (260 to 270 kbp) and the gene associated with pyocyanin production (located between 90 and 130 kbp). The genome of environmental isolate p3 contained the highest number of virulence genes among the environmental isolates, mainly corresponding to the *wbpB, wbpG, wbpH, wbpI, wbpJ*, and *wbpK* genes, located between 230 and 240 kbp, and the *wzy* gene, located between 30 and 40 kbp. These genes encode for proteins associated with lipopolysaccharide biosynthesis [42].

### 3.5. Comparison of Genes Associated with Antibiotic Resistance

**Figure 2:**
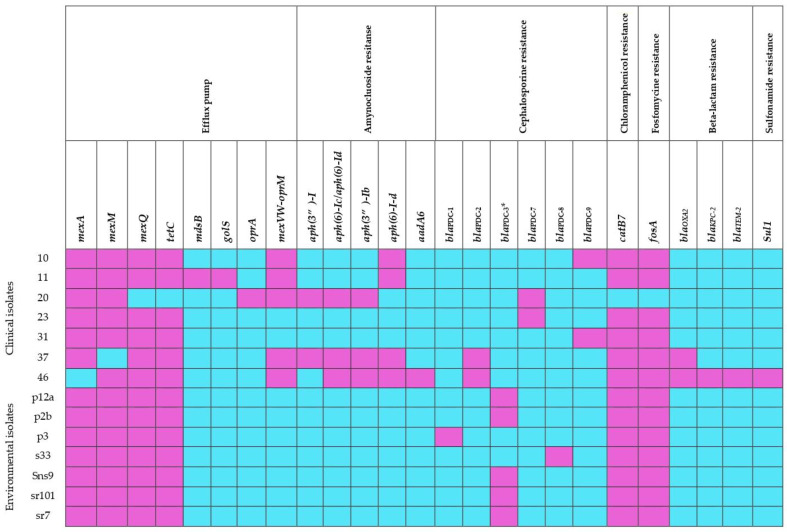
Heat map representing the absence (aquamarine blue color) and the presence (purple color) of genes that encode antimicrobial resistance determinants in environmental and clinical isolates of *P. aeruginosa*. * statistically significant differences, *p*-value = 0.0475.

A total of 135 genes associated with antimicrobial resistance in clinical and environmental isolates of *P. aeruginosa* were collected (Supplementary Material S2), of which 72.59% (98 genes) were common among the genomes analyzed. These genes are related to antibiotic target resistance mechanisms, efflux pumps, changes in membrane permeability, antibiotic inactivation, and antibiotic target modification (Table 4). The remaining 27.41% (37 genes) were different; these most significant differences are shown in the heat map in Figure 2, where it can be seen that clinical isolates have a greater number of resistance genes than environmental isolates.

The identified genes include those associated with efflux pumps, resistance to aminoglycosides, cephalosporins, fosfomycin, chloramphenicol, sulfonamides and beta-lactamases.

The genes responsible for efflux pumps, namely *mexA, mexM, mexQ* and *tetC*, were present in all environmental isolates and in four of the clinical isolates. Clinical isolate 11 is noteworthy for having the *mdsB* genes, which encode a membrane antiporter protein of MexPQ-OpmE pump, and the *golS* gene, which is a regulator of the MerR family of efflux pumps. However, this isolate was typed as susceptible to all antibiotics used. It is worth noting that the *mexVW-oprM* gene, related to efflux pumps, was found in all clinical isolates but was absent in environmental isolates.

The analysis of environmental isolates revealed the absence of aminoglycoside modifier genes, whereas clinical isolates presented at least one. All the genomes analyzed, with the exception of clinical isolate 20, presented the *catB7* and *fosA* genes, which are associated with resistance to chloramphenicol and fosfomycin, respectively.

The *bla*_OXA-2_ gene was exclusively observed in clinical isolates 37 and 46 MDR. Isolate 37 was resistant to almost all antibiotics tested, including cephalosporins such as cefepime and ceftazidime, ciprofloxacin, amikacin, piperacillin/taxobactam, and carbapenems such as imipenem and meropenem, and isolate 46 showed resistance to all antibiotics tested. Of particular interest were the *bla*_KPC-2_ and *bla*_TEM-2_ genes, which encode for extended spectrum β-lactamases. Additionally, this same isolate presented the *aadA6* and *sul1* genes associated with resistance to aminoglycosides and sulfonamides, respectively. Isolates 23 and 31 were found to be MDR despite the absence of previously described genes. However, they were found to contain genes encoding for Pseudomonas-derived cephalosporinase (PDC) β-lactamase-encoding gene, such as *bla*_PDC-*7*_ and *bla*_PDC-9_, respectively.

Additionally, the following cephalosporin-resistant genes were found in clinical isolates: *bla*_PDC-2_, *bla*_PDC-7_, and *bla*_PDC-9_. In contrast, environmental isolates contained exclusively the *bla*_PDC-1_, *bla*_PDC-*3*_, and *bla*_PDC-8_ genes. In particular, only the *bla*_PDC-3_ gene showed statistically significant differences (*p* = 0.0475) and was detected exclusively in environmental isolates. The identification of this PDC and its polymorphisms have been linked to resistance to B-lactam antibiotics [43].

## 4. Discussion

*P. aeruginosa* isolates from different environmental sources (soil and DWTP) showed different susceptibility patterns, suggesting possible differences in antibiotic resistance mechanisms in these settings, while clinical isolates showed worrisome resistance patterns that may reflect the prevalence of antibiotic resistance in clinical settings. Resistance to ciprofloxacin and aztreonam was most frequently observed. Other researchers observed that *P. aeruginosa* isolates can exhibit varying levels of resistance to different classes of antibiotics, which can have implications for the treatment of infections caused by this bacterium [44,45,46,47].

The MLST results provide information on the genetic diversity and distribution of *P. aeruginosa* isolates, highlighting that HRCs were found exclusively in clinically derived isolates, suggesting a possible link between clinical setting and virulence [10,11,12,45]. Specifically, isolates 37 and 46 are ST235 with an MDR profile, and isolate 11 is ST111 with a sensitive profile. These isolates will impact human medical epidemiology due to their capacity for dissemination and persistence. When evaluating the genome, we identified several resistance genes associated with gene insertion elements, which should contain an accessory genome, but these were not identified and absent in the environmental isolates included in this study.

Within isolated environmental samples, it was observed that MDR microorganisms are present in environments such as wastewater treatment plants, as was the case for p3, which showed resistance to aztreonam, ciprofloxacin, and amikacin. However, no ST associated with HRC was found, which is a finding that has been previously described in other studies both inside and outside of Colombia [48,49,50,51]. These studies highlight the dissemination of microorganisms through water sources, which could spread bacterial resistance. The diversity of ST, including those associated with non-HRC, highlights the complexity of *P. aeruginosa* epidemiology and the potential variation in virulence and antibiotic resistance profiles among clinical isolates. Environmental isolates exhibited a distinct ST pattern, but none were linked to HRC, indicating a different genetic background or evolutionary history compared to clinical isolates.

The general characteristics of the genome size (6.2 to 7.0 Mbp) and number of CDs (5841 to 6488) of both clinical and environmental isolates of *P. aeruginosa* were within the ranges reported for this species [52,53]. However, when comparing these values with those reported for the PAO1 reference strain (6.2 Mbp and 6200 CDS), it is evident that the clinical isolates have a slightly larger size than the environmental isolates, which present similar values to this strain. These data confirm that clinical isolates present greater genetic information, which may be due to the presence of plasmids and genetic elements not evaluated in this research, but which has been confirmed in similar research [17] due to the acquisition of genes in the environment where they often live.

Regarding the genes that encode virulence factors in clinical and environmental isolates, similarities were found in 62%, which is a result that suggests that *P. aeruginosa* intrinsically possesses a repertoire of genes that encode a wide variety of virulence determinants but that it also acquires additional genetic information [12,54]. These findings can be compared with studies such as the one by Wolfgang and colleagues in which the analysis of the genome composition of a collection of 18 strains of *P. aeruginosa* of different origins was carried out using a whole-genome DNA microarray [55]. A high level of conservation of virulence genes (≈97%) was found in clinical strains, and that finding extends to environmental isolates, similar to what was found in this study, which suggests that environmental isolates have genetic information that is conserved and shared with clinical isolates [55].

In this research, it was observed that the largest number of virulence genes found for both clinical and environmental isolates was that associated with the mechanism of adhesion and motility (85 genes). This result is largely consistent with what was found by Nain and Karim [6], and that allows justifying the high adherence potential of *P. aeruginosa*, thanks to the presence of genes for the synthesis of compounds such as alginate and extracellular structures such as flagella and pili. These qualities allow it to invade various environments, from the simplest to the most difficult and complex, where it later colonizes and can cause disease in animals and humans [56].

Other significant genes identified include those encoding secretion systems. A total of 76 genes were identified, five of which were described in *P. aeruginosa* (T1SS, T2SS, T3SS, T5SS, and T6SS). These systems modulate the immune response of hosts by releasing several proteins, toxins, and enzymes [57,58]. However, this study found that all isolates, except for clinical isolate 20, possess the T2SS, T3SS, and T6SS secretion systems. Isolate 20 lacks the genes associated with T3SS, which are located between 90 and 130 kbp (Figure 1), making it an atypical isolate similar to the reference strain PA7. PA7 also lacks most of the genes associated with this secretion system and, in general, with virulence. However, both isolates possess significant genetic information associated with antimicrobial resistance acquired by gene transfer elements [41]. Isolate 20 lacks T3SS-associated genes but possesses *exlA* and *exlB* genes, which encode toxins capable of inducing plasma membrane rupture in host cells [55]. These genes are associated with virulence related to cytolytic action, which is characteristic of those isolates that are considered atypical [59]. Of particular interest, this isolate does not present an HRC-associated ST (ST1978) and exhibits phenotypic resistance to ciprofloxacin (fluoroquinolone). In this study, we did not identify any gene transfer elements that could shed light on the genetic characteristics; further investigation is necessary.

The major clinical isolates MDR were found to contain the *exoU* gene, which is related to T3SS and showed statistically significant differences. This gene is located in pathogenicity island 2 (PAPI-2) and encodes the ExoU protein, which is activated by host ubiquitin (Ub). Upon activation, ExoU causes destructive activity on the membrane, resulting in host cell death [60,61]. This suggests that the clinical isolates studied are cytotoxic and have a high capacity to cause serious epithelial lesions. The exclusive predominance of ExoU in this type of isolate indicates that this gene contributes to the pathogenicity of the clinical isolates studied [53]. The presence of the *exoU*-coding gene was detected in only one environmental isolate, p12a, which likely originated from domestic wastewater sources that converge in the DWTP of the municipality where the sample was taken. This isolate exhibited a phenotype sensitive to all the antimicrobials evaluated. In contrast, the isolate from the same collection site, p3, with an MDR profile has a genotype (*exoU* negative), suggesting that the *exoU*-positive genotype is not necessarily associated with the presence of phenotypic resistance, but rather with virulence, as previously described [62,63].

In contrast, genes associated with the siderophore secretion mechanism predominated in the environmental isolates. *P. aeruginosa* secretes siderophores such as pyoverdine under conditions of iron deficiency, which is a necessary aspect for the survival of this organism [8,64]. At a clinical level, it is essential to capture iron from cells of the host where the bacterium uses it in the infection stage. Xie and colleagues reported that the deficiency or absence in the production of siderophores reflects a reduction in virulence in *P. aeruginosa* [65]. In the environmental isolates studied, this type of gene predominated, which is a finding that is important at the biotechnological level since it can be used in the bioremediation of heavy metals and metalloids. Once chelated, metals and metalloids can be sequestered through different mechanisms, such as bioadsorption and bioaccumulation, thus allowing the removal of contaminants from the environment [66]. Based on these results, it is inferred that in humans, they could have a characteristic associated with virulence and be involved in the development of infections, but at an environmental level, it is a survival mechanism. Variations in the presence of these genes suggest an effect on the release of pyoverdine [67]. This raises questions about the role of the enzymes encoded by these genes in the transport and export of pioverdine.

This study found genes associated with pyocyanin synthesis in all environmental and clinical isolates except isolate 20, which may indicate a deficiency in the cytotoxic and regulatory functions of the QS system [68,69]. This finding also suggests a deficiency in virulence in this isolate, which requires further genomic studies to determine the origin of these differences.

However, in all isolates, both clinical and environmental, the presence of QS-associated genes was evidenced, which in *P. aeruginosa* are important in its virulence due to their function in intercellular communication that allows the adaptation of a bacterial population to microenvironmental changes by the diffusion of small molecules called autoinducers or acyl-homoserine lactones (AHLs) through the bacterial membrane [8]. QS genes activate other genes related to various virulence factors, such as *lasB*, which encodes elastases, and the *rhlAB* operon, which determines the production of rhamnolipids [8]. In *P. aeruginosa,* the systems of detection of QS, *lasI/lasR* and *rhlI/rhlR* [57,58], and the presence of the genes that encode them were also found in this investigation.

Another predominant virulence factor among the *P. aeruginosa* isolates was rhamnolipid (RL) biosynthesis, represented by the *rhlA*, *rhlB* and *rhlC* genes, present in all the isolates analyzed, except that the *rhlC* gene was absent in clinical isolate 20. The *rhlA* and *rhlB* genes are organized in the rhlAB operon, while rhlC is in a different region of the genome, which is associated with a deficiency in di-rhamnolipid production and an alteration of swarming mobility patterns [70]. In clinical terms, it has been shown that *P. aeruginosa* requires RL production to invade the respiratory epithelium [71]. At a physiological level, they are essential for the growth of *P. aeruginosa* on hydrophobic substrates, and they are also important for the colonization of various habitats since they are capable of reducing surface tension, forming emulsions, and causing the pseudo-solubilization of insoluble substrates. These characteristics allow the production of rhamnolipids to be used in hydrocarbon bioremediation processes, both aliphatic and aromatic, as well as heavy metals such as lead and cadmium [71,72]. The absence of any of these genes leads to a deficiency in the enzymes involved in the production of rhamnolipids related to various functions in the natural environment.

The environmental isolate p3 evidenced, unlike the other isolates, showed the presence of the genes *wbpB*, *wbpG*, *wbpH*, *wbpI*, *wbpJ*, *wbpK* and *wzy* which encode for one of the components of the lipopolysaccharides, which is called O-antigen [42]. Two types of O-antigen are observed in *P. aeruginosa*: the A-banded O-antigen, which is common to most strains, and the B-banded O-antigen, which is variable and encoded by the above genes. According to the literature, the variability of the B band confers serogroup specificity, making it suitable for international antigenic typing [73,74].

It should be noted that although it cannot be predicted in this study whether the detected genes are functional, it is evident that regardless of their origin, the *P. aeruginosa* isolates conserve a large group of genes that encode different virulence determinants; therefore, it can be inferred that extra-hospital isolates are a potential reservoir of genes of interest for the health of humans and animals. However, it should be considered that the infection success of this pathogen also depends on the conditions of the host, such as being hospitalized or immunocompromised.

The variety of virulence determinants in *P. aeruginosa* is crucial for the colonization of diverse ecosystems beyond their role in the infection of human hosts, which indicates that some of these pathogenicity mechanisms can adapt to fluctuating conditions depending on their environmental requirements [75,76]. It is evident that when this species is used in processes such as the bioremediation of hydrocarbons and other contaminants, biosafety protocols must be stipulated and regulated to minimize the health risk in the environments where they are used.

Regarding the comparison of genes associated with antimicrobial resistance, this research shows that the environmental and clinical isolates of *P. aeruginosa* present 72.59% similarity (Table 3) but also differ in various groups of genes, reaffirming the great genetic plasticity of this bacterium [77].

The percentage of similar genes between isolates is possibly because *P. aeruginosa* possesses a high level of intrinsic resistance to various antibiotics through mechanisms such as restricted outer membrane permeability, efflux systems that pump antibiotics out of the cell, and variable encoding of AmpC-like enzymes that inactivate antibiotics [78,79]. The study identified different variables of cephalosporinases, including Pseudomonas-derived cephalosporinase (PDC) or AmpC-type β-lactamases, which are normally produced by *P. aeruginosa*.

These variables confer resistance to penicillins and second- and third-generation cephalosporins [80,81]. Castanheira and colleagues reported that the overexpression of chromosomal cephalosporinase (PDC) generates resistance to most cephalosporins and, to a lesser extent, to imipenem [82]. Statistically significant differences were found in the *bla*_PDC-*3*_ gene, which was identified in a greater proportion in environmental isolates and encodes *bla*_PDC-3_, except for the isolate p3 where *bla*_PDC-1_ was identified, which encodes *bla*_PDC-1_ and was also found in the reference strain PAO1. The PDC-1 and PDC-3 proteins differ by a change in the amino acid at position 79 [83]. Furthermore, other isolates within this group exhibit variations in a maximum of two amino acids when compared to PAO1 [84]. Clinical isolates, on the other hand, have different PDCs than environmental isolates. As previously reported, environmental isolates have variations in two to five amino acids [85]. These findings support the idea that the PDC-encoding gene is conserved with minimal variations, suggesting that its functions may be similar in relation to antibiotics [43]. However, the polymorphism in the PDC sequence requires further investigation.

Among the differences found, it can be seen in Figure 2 that the clinical isolates have a greater quantity and diversity of genes, which are mainly associated with expulsion pumps, the inactivation of antibiotics, and resistance to carbapenems, aminoglycosides and sulfonamides. Many of these mechanisms of resistance are usually acquired from mobile genetic elements [58,86].

Additionally, in this study, the *mexVW-oprM* gene was found exclusively in all clinical isolates regardless of their susceptibility profile, which is consistent with that reported for *P. aeruginosa*, which is associated with resistance to multiple antibiotics such as chloramphenicol, fluoroquinolones, and macrolides, among others [87,88,89]. Likewise, the *oprA, golS*, and *mdsB* genes were found exclusively in clinical isolates. These genes are associated with a resistance mechanism that consists of the generation of a pump that expels antimicrobials out of the bacteria. The *golS* and *mdsB* genes (found in the sensitive clinical isolate 11) associated with the MdsABC ejection pump have not been reported in *P. aeruginosa* [90,91], but they have been reported in species of the genus *Salmonella*, so more detailed studies are required to identify the mechanisms by which this clinical isolate could have acquired these genes.

Clinical isolate 20 was found to contain the *oprA* gene, which encodes for an outer membrane channel. According to Morita and colleagues, this gene is uncommonly found in strains of this species [92]. However, when present, it may functionally cooperate with the *mexXY* operon, since it does not have a specific gene for the outer membrane channel. It is noteworthy that the *mexXY*-mediated efflux pump is a significant determinant of aminoglycoside resistance only in *P. aeruginosa*.

Additionally, only in clinical isolates was the presence of the genes *aph(3″)-I*, *aph(6)-Ic/aph(6)-Id*, *aph(3″)-Ib*, and *aph(6)*- identified; these genes encode aminoglycoside-modifying enzymes, consistent with what has been reported in other studies, being favored by the selective pressure exerted in hospital environments due to the constant use of these antibiotics [93,94], which explains why they were not found in environmental isolates.

Clinical isolate 46, which has a multidrug-resistant (MDR) profile, carries the *aadA6* gene that confers resistance to aminoglycosides, including streptomycin and spectinomycin; this finding is consistent with the results reported by Papadovasilaki and colleagues, who detected the *aadA6* gene in an MDR clinical isolate of *P. aeruginosa* Ps100 located in the integron In118 [95]. This isolate also contains the *sul1* gene, which is associated with sulfonamide resistance and is frequently found in species of the *Enterobacteriaceae* family. However, Ruiz and colleagues reported that this gene is also commonly found in clinical isolates of *P. aeruginosa*, as there is a high correlation between the presence of a class 1 integron and resistance to sulfamethoxazole [96]. Based on these findings, the clinical isolate MDR 46 can be considered a reservoir of resistance genes of high clinical interest. Its genomic profile (*exoU* positive) suggests that it could be a highly virulent superbug. Detailed investigations are required to identify the mobile genetic elements present in its genome and the dissemination mechanisms associated with these genes.

Another one of the main differences found was the presence of the ${bla}_{KPC-2}$ and ${bla}_{TEM-2}$ genes only in the hospital isolate MDR 46, genes that according to the literature are acquired from plasmids and that confer resistance to carbapenems and cefalosporins, respectively [97,98,99], which are described in clinical isolates of *P. aeruginosa* with phenotypes similar to those of this isolate [100]. Carbapenemases are enzymes that hydrolyze carbapenem antibiotics and represent the most versatile family of β-lactamases. According to previous studies, the rapid spread of KPC enzymes has been linked to the presence of this type of gene through the Tn4401 mobile genetic element and its isoforms. In Colombia, *bla*_KPC-2_ is found more frequently in clinical strains associated with the Tn4401b isoform [100,101,102], which, in turn, is associated with the IncP-6 and IncU plasmids, facilitating the transmission of resistance mechanisms to different types of bacteria of the same species in *P. aeruginosa* as in species of the *Enterobacteriaceae* family [7].

On the other hand, the group of *bla*_TEM-2_ genes found in the MDR 46 isolate confers resistance against third-generation cephalosporins through the production of extended-spectrum β-lactamases (ESBLs), another of the most important resistance mechanisms in *P. aeruginosa* [103,104], found in various regions worldwide, complicating the therapy of infections caused by ESBL-producing bacteria [105]. The *bla*_OXA-2_ gene, identified in two MDR isolates (46 and 31), also encodes ESBL with a high spectrum of hydrolytic activity against cloxacillin and oxacillin and little inhibition by clavulanic acid [106]. These findings confirm its phenotypic expression and the pressure exerted by the clinical environment.

In the environmental isolates sr101 and sr75 with a sensitive phenotype, the *tetC* gene, which encodes the Tet protein, was identified; this gene is associated with the membrane protein in charge of expelling tetracyclines, indicating the ability of *Pseudomonas* to resist this antibiotic [107].

As reported by Martínez and colleagues, the intrinsic resistance possessed by environmental isolates of opportunistic pathogens such as *P. aeruginosa* is of great importance for the colonization of the rhizosphere, a habitat that may contain toxic compounds produced by plants or the associated microbiota, as well as the colonization of a great diversity of habitats [85]. By sharing a large part of these resistance genes with hospital isolates, it is proposed that the intrinsic resistance phenotype presented by some opportunistic pathogens of environmental origin was acquired during the evolution of these microorganisms long before the discovery of antibiotics [108]. On the other hand, environmental and hospital isolates of *P. aeruginosa* have different genes. This is possibly because clinical isolates favor the selection and spread of antibiotic resistance by inappropriate and indiscriminate use, thus generating selective pressure in hospital environments [108,109].

## 5. Conclusions

In this study, it was evidenced that the clinical isolates of *P. aeruginosa* presented a greater number and diversity of genes associated with resistance to antibiotics and that regardless of their origin, they possess a wide arsenal of genes that confer multiple mechanisms of virulence and resistance to antibiotics, which explains why it is considered one of the most important pathogenic bacteria worldwide. According to the results of this research, it can be affirmed that most virulence determinants provide *P. aeruginosa* with the ability to survive and colonize different hosts and ecosystems, as confirmed by evidence in its genome. Therefore, it can be inferred that extra-hospital isolates are a potential reservoir of genes of interest for human and animal health. On the other hand, this study showed that although hospital isolates share a large number of genes associated with antimicrobial resistance with environmental isolates, they also differ in a wide group of genes, the latter possibly due to the selective pressure exerted in hospital environments due to the constant use of antibiotics. In this study, no medically important resistance genes associated with MDR phenotypes were identified in the environmental isolates, so it is theorized that the environment from which they were isolated leads to wild-type phenotypes. Therefore, more comparative research is required to elucidate the current situation of the emergence and spread of resistance to antibiotics and of gene transfer mechanisms not only in hospital environments but also in natural environments.

## Figures and Tables

**Table 1:** Origin of the clinical and environmental isolates of *P. aeruginosa*.

Clinical Isolates		Environmental Isolates	
ID	origin	ID	origin
10	Acute clinical infection/not identified	p12a	Water, inlet of the facultative lagoon of the domestic wastewater treatment plant.
11	Acute clinical infection/blood sample	p2b	Water, outlet of the anaerobic lagoon of the domestic wastewater treatment plant
20	Acute clinical infection/blood sample	p3	Water, outlet of the facultative lagoon of the domestic wastewater treatment plant.
23	Acute clinical infection/blood sample	s33	Soil, sugarcane crop, located south of Cali.
31	Acute clinical infection/respiratory sample	sn99	Soil, Experiment station of the Valle University
37	Acute clinical infection/not identified	sr101	Soil, riverbank Pance
46	Acute clinical infection/gastrointestinal sample	sr75	Soil, riverbank Pance

**Table 2:** Antibiotic susceptibility profile of *P. aeruginosa* from clinical and environmental isolates.

Source	Isolate	Monobact	Cephalosporin		Fluoroq	Carbapenems		Comb β-lact	Aminogluc
		AZT	CPE	CAZ	CP	IMP	MER	P/T	AK
Water	p12a	S	S	S	S	S	S	S	S
	p2b	S	S	S	S	S	S	S	S
	p3	R	S	S	R	S	S	S	R
Soil	s33	R	S	S	S	S	S	S	S
	sn99	S	S	S	S	S	S	S	S
	sr101	S	S	S	S	S	S	S	S
	sr75	S	S	S	S	S	S	S	S
Hospital environment	37	S	S	R	R	R	R	R	R
	46	R	R	R	R	R	R	R	R
	31	R	R	R	R	R	R	R	R
	23	R	S	R	R	R	R	R	S
	20	S	S	S	R	S	S	S	S
	10	S	S	S	S	S	S	S	S
	11	S	S	S	S	S	S	S	S

Conventions: antibiotics: aztreonam (AZT), cefepime (CPE), ceftazidime (CAZ), ciprofloxacin (CP), imipenem (IMP), meropenem (MER), piperacillin/clavulanic acid (P/T) and amikacin (AK). Interpretation: sensitive (S) and resistant (R).

**Table 3:** Genetic characteristics of *P. aeruginosa* clinical and environmental isolates.

Source	ID	Genome Length (Mbp)	CD	Content of G + C (%)	MLST
Clinic	10	6.9	6633	66.0	253
	11	7.0	6845	66.0	111
	20	6.3	6087	66.6	1978
	23	6.4	6017	66.5	3236
	31	6.8	6535	66.0	253
	37	6.6	6406	66.0	235
	46	6.7	6488	66.1	235
	Average	6.67	6430	66.2	
Water	p12a	6.5	6099	66.2	1427
	p2b	6.3	5949	66.5	1993
	p3	6.2	5860	66.6	549
Soil	s33	6.2	5841	66.6	3579
	sn99	6.2	5892	66.5	252
	sr101	6.3	5890	66.5	282
	sr75	6.2	5907	66.5	282
	Average	6.27	5920	66.5	

**Table 4:** Genes associated with antimicrobial resistance among the environmental and hospital isolates of *P. aeruginosa* common to all isolates.

Resistance Mechanism	Genes
Target antibiotic	*alr*, *ddl*, *dxr*, *EF-G*, *EF-Tu*, *folA*, *dfr*, *folP*, *inhA*, *fabI iso-tRNA*, *kasA*, *murA*, *rho*, *rpoC*, *s10p*, *rpoB.*
Efflux pump	*macA*, *macB*, *mdtABC-OMF*, *mdtABC-tolC*, *mexAB-oprM mexCD-oprJ*, *mexEF-OprN*, *mexHI-opmD*, *mexJK-oprM/opmH. mexPQ-opmE*, *mexXY-oMP*, *tolC/opmH*, *triABC-opmH*, *amrA*, *amrB*, *mexB*, *mexC*, *mexD*, *mexE*, *mexF*, *mexG*, *mexH*, *mexI, mexJ, mexK, mexL, mexN, mexP*, *mexR*, *mexS*, *mexV, mexW, mexZ, nalC, nalD, nfxB*, *opmD*, *opmE*, *opmH, oprJ, oprM, oprN, phoP, phoQ, triA*, *triB*, *triC*, *emrE.*
Changes in membrane permeability	*occD2/opdC*, *occD3/opdP*, *occD4/opdT*, *occD5/opdI*, *occD6/oprQ*, *occD7/opdB*, *occD8/opdJ*, *occK1/opdK*, *occK10/opdN*, *occK11/opdR*, *occK3/opdO*, *occK4/opdL*, *occK5/opdH*, *occK6/opdQ*, *occK8/oprE*, *occK9/opdG*, *oprB*, *porF*, *oprD*, *pgsA*, *pmrA*, *pmrB. gdpD.*
Antibiotic inactivation	*aph(3′)-II/aph(3′)-XV*, *bla_OXA-50_ aph(3′)-IIb.*
Antibiotic target modification	*gyrA*, *gyrB*, *parC*, *parE.*

## Data Availability

Data supporting the results of this research can be found in: https://www.ncbi.nlm.nih.gov/sra/PRJNA1075801 (accessed on 12 February 2024).

## Supplementary Materials

The following supporting information can be downloaded at https://www.mdpi.com/article/10.3390/microorganisms12061116/s1, Table S1: Virulence genes found in clinical and environmental isolates of *P. aeruginosa*; Table S2: Resistance genes found in clinical and environmental isolates of *P. aeruginosa*.
